# Pulmonary vascular changes in extremely preterm sheep after intra-amniotic exposure to *Ureaplasma parvum* and lipopolysaccharide

**DOI:** 10.1371/journal.pone.0180114

**Published:** 2017-06-30

**Authors:** Monique G. M. Willems, Matthew W. Kemp, Laura A. Fast, Nick M. M. Wagemaker, Leon E. W. Janssen, John P. Newnham, Matt S. Payne, Owen B. Spiller, Suhas G. Kallapur, Alan H. Jobe, Tammo Delhaas, Boris W. Kramer, Tim G. A. M. Wolfs

**Affiliations:** 1 Department of Pediatrics, Maastricht University Medical Center, Maastricht, The Netherlands; 2 Department of BioMedical Engineering, Maastricht University Medical Center, Maastricht, The Netherlands; 3 GROW School for Oncology and Developmental Biology, Maastricht University Medical Center, Maastricht, The Netherlands; 4 School of Women's and Infants' Health, The University of Western Australia, Perth, Western Australia, Australia; 5 School of Medicine, Cardiff University, University Hospital of Wales, Cardiff, United Kingdom; 6 Division of Neonatology, Cincinnati Children's Hospital Medical Center, Cincinnati, Ohio, Unites States of America; 7 CARIM School for Cardiovascular Diseases, Maastricht University Medical Center, Maastricht, The Netherlands; 8 School for Mental Health and Neuroscience, Maastricht University, Maastricht, The Netherlands; Hopital Robert Debre, FRANCE

## Abstract

**Background:**

Chorioamnionitis can induce pulmonary inflammation and promote bronchopulmonary dysplasia development, distinguished by alveolar simplification and impaired vascular growth. Chorioamnionitis is more common during the extremely preterm canalicular lung stage (crucial for vascular development); and increases the risk for subsequent sepsis. We hypothesized that single/combined exposure to chronic and/or acute inflammation induces pulmonary inflammatory responses and vascular changes.

**Methods:**

Ovine fetuses were intra-amniotically exposed to chronic *Ureaplasma parvum* (UP) at 24 days (d) before extreme preterm delivery at 94d (term 147d) and/or to lipopolysaccharide (LPS) 7 or 2d before delivery. Pulmonary inflammation, vascular remodeling and angiogenic factors were assessed.

**Results:**

LPS exposure increased CD3-positive and myeloperoxidase-positive cells. Combined UP-LPS exposure increased pulmonary inflammation compared with 2d LPS or UP groups. The UP+2d LPS group had an increased adventitial fibrosis score when compared with UP-treated animals. A reduced wall-to-lumen ratio was found in the 7d LPS animals when compared to the 2d LPS-treated animals. Exposure to UP+2d LPS reduced *VEGF* and *VEGFR-2* levels compared with 2d LPS-treated animals. Angiopoietin-1 (*Ang1*) and tunica interna endothelial cell kinase 2 (*Tie-2*) levels were decreased after UP+7d LPS as well as after 7d LPS, but not with UP alone.

**Conclusion:**

Chronic UP and subsequent LPS exposure increased pulmonary inflammation and decreased expression of angiogenic growth factors and receptors when compared to single hit-exposed animals.

## Introduction

Preterm birth is frequently associated with chorioamnionitis, an inflammation of the chorion, amnion and placenta [1, 2]. Direct pulmonary contact with contaminated amniotic fluid can initiate pulmonary inflammation [3, 4]. Dependent on the presence of additional postnatal factors such as ventilation and sepsis, a progressive pulmonary inflammatory response may be induced which promotes the development of bronchopulmonary dysplasia (BPD) [5]. BPD is characterized by alveolar simplification and impaired vascular growth [6, 7]. In addition, BPD patients can display structural vascular alterations and decreased expression of angiogenic growth factors and corresponding receptors, such as vascular endothelial growth factor (VEGF) [8, 9]. Increasing evidence suggests that pulmonary vascular development is very important during lung growth [10]. The vascular hypothesis implies that disruption of pulmonary angiogenesis during essential stages of fetal growth may impair alveolarization and ultimately contribute to the pulmonary hypoplasia that typifies BPD [10, 11]. In neonatal rats it was demonstrated that anti-angiogenic agents including VEGF (receptor) inhibitors attenuated pulmonary vascular growth and decreased alveolarization, which can persist until adulthood [12, 13].

Intra-amniotic infection is often polymicrobial, with *Ureaplasma parvum* (*U*. *parvum*) as one of the most common microorganisms detected in amniotic fluid of women with preterm prelabor rupture of membranes (PROM) and in respiratory aspirates of preterm infants [14, 15]. Respiratory colonization of *Ureaplasma* is associated with the development of BPD in neonates [16]. In addition, *U*. *parvum* has been shown to selectively down regulate antimicrobial peptide expression, which may increase the susceptibility to additional microorganisms *in utero* [17]. Viral infections can sensitize pregnant mice to a subsequent bacterial infection as well, resulting in preterm delivery and fetal death [18]. In our translational ovine chorioamnionitis model, we have previously not only shown that antenatal inflammation can induce fetal pulmonary inflammation and vascular changes, but also that chronic intra-amniotic exposure to *U*. *parvum* suppressed fetal pulmonary responsiveness to a subsequent endotoxin stimulus and thus renders the fetus more susceptible to an additional inflammatory stimulus [19–21]. Nevertheless, our studies were performed using fetal sheep born at 120–124 days of gestational age (dGA) which is comparable to approximately 30 weeks of human gestation [22]. It is unknown how the fetal pulmonary system will respond to exposure to chronic *U*. *parvum* and subsequent endotoxin at a lower gestational age. The incidence of histologic chorioamnionitis is higher in infants born at a lower gestational age (reaching up to 66% at 20–24 weeks of gestation) and sepsis occurs more frequently in very low birth weight infants when exposed to chorioamnionitis [23, 24]. Furthermore, extremely preterm infants born during the canalicular stage of lung development are in a crucial phase of vascular lung development [6, 10]. Consequently, the aim of this study was to determine whether intra-amniotic chronic *U*. *parvum* exposure or acute inflammation during canalicular lung development induced a pulmonary inflammatory response and concomitant vascular changes. In addition, we determined whether chronic pre-exposure to *U*. *parvum* modulated this pulmonary response in the presence of a subsequent acute inflammatory stimulus. We hypothesized that single or combined exposure to chronic *U*. *parvum* and/or acute inflammation provoked a pulmonary inflammatory response and induced vascular changes. We exposed extremely preterm sheep intra-amniotically to chronic *U*. *parvum* and/or lipopolysaccharide (LPS) and assessed inflammatory parameters, vascular remodeling and expression levels of several angiogenic growth factors and their receptors in the ovine fetal lungs at the canalicular stage. In the current study, we have shown that in extreme preterm sheep contamination of the amniotic fluid mounted a pulmonary inflammatory response with concomitant impairment of the vasculature. In addition, we have shown that combined exposure to *U*. *parvum* and LPS enhanced pulmonary inflammation and decreased expression of angiogenic growth factors and receptors when compared to UP or LPS exposed animals.

## Methods

### Animal model & sampling procedure

The study was carried out in accordance with the ‘Animal Welfare Act (2002)’ and the requirements of the ‘Australian code for the care and use of animals for scientific purposes (8th edition, 2013)’. All animal experiments were approved by the Animal Ethics Committee of The University of Western Australia (reference number RA/3/100/312) and were performed at The University of Western Australia (Perth, Australia). The human endpoints for this study included irresponsive pain, shock and preterm labor. In case a human endpoint would be reached, the animal would be euthanized after consultation with a practicing veterinarian who is experienced in treating ruminants. However, during the time of the experiment, no animals died before reaching the end of the experiment. Animal wellbeing was daily monitored. To minimize suffering and distress, animals were kept in flock and ultrasound-guided injections were performed by staff experienced in delivering these injections.

**Fig 1:**
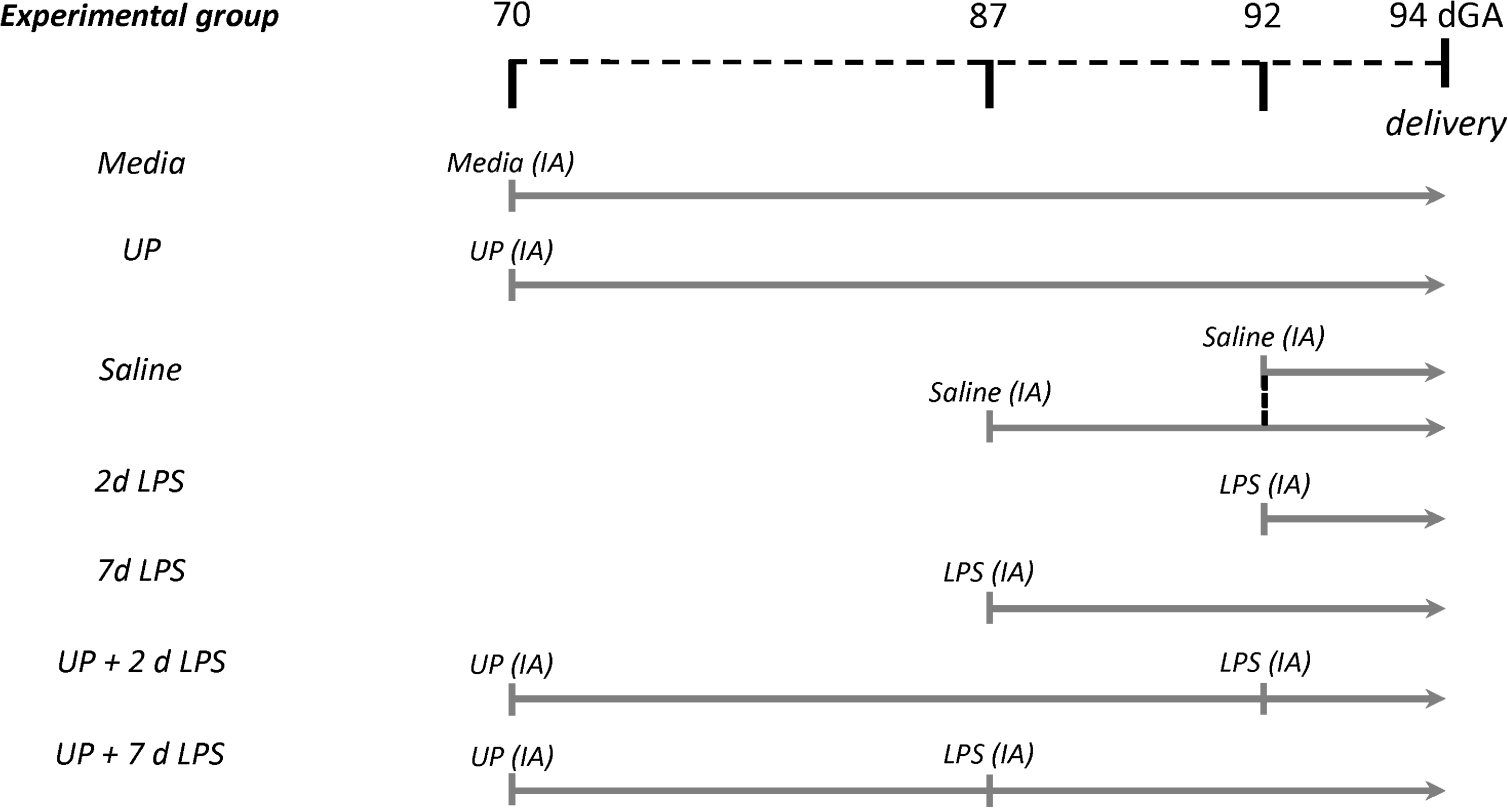
Experimental study design. Ovine fetuses were randomized and prematurely delivered at 94 days of gestational age (dGA) (term at ±147 dGA). In the ‘UP’ group, animals received an intra-amniotic (IA) injection with *U*. *parvum* (UP) 24 days prior to preterm birth. In the ‘2d LPS’ and ‘7d LPS’ groups, animals were IA injected with lipopolysaccharide (LPS) 2 or 7 days before preterm delivery. Control animals received media 24 days (‘Media’ group) or saline 2 or 7 days (‘Saline’ group) before preterm birth. To assess a potential effect of *U*. *parvum* on subsequent exposure to LPS, two separate groups of animals received IA *U*. *parvum* 24 days before delivery followed by either IA LPS for 2 days (‘UP+2d LPS’ group) or for 7 days (‘UP+7d LPS’ group) before preterm birth.

Date-mated Merino ewes (*Ovis aries*) were randomized to receive ultrasound-guided intra-amniotic (IA) injections of 2 x 10^5^ color-changing units (CCU) *U*. *parvum* serovar 3 at 24 days prior to preterm birth (‘UP’ group); or 10 mg LPS (*Escherichia coli* 055:B5; Sigma-Aldrich, St Louis, MO) diluted in 2 mL saline at 2 or 7 days before preterm delivery (‘2d LPS’ and ‘7d LPS’ groups) (**Fig 1**). Control animals received media 24 days (‘media’ group) or saline 2 or 7 days (‘saline’ group) before preterm birth. To assess a potential effect of *U*. *parvum* on subsequent exposure to LPS, two separate groups of animals received IA *U*. *parvum* 24 days before delivery followed by IA LPS 2 days (‘UP+2d LPS’ group) or 7 days (‘UP+7d LPS’ group) before preterm birth. All fetuses (n = 6–8 per group) were prematurely delivered at 94 days of gestational age (dGA) by Caesarean section (term at ±147 dGA). Following euthanasia of the ewe using intravenous pentobarbital, the fetus was delivered and euthanized. Lung tissue from the right lower lobe (RLL) was snap-frozen until further analysis. The right upper lobe (RUL) was inflation-fixated at 30 cm H_2_O pressure in 10% buffered formalin, administered through the bronchus, for 24 hours and processed for paraffin embedding. Vessels were not fixated separately.

### Immunohistochemistry

Paraffin-embedded RUL tissues were cut (4 μm) and stained for CD3 to identify T cells, for myeloperoxidase (MPO) to detect activated neutrophils and monocytes, and for α-smooth muscle actin (α-SMA) to visualize the tunica media of arterioles [20, 25]. Information about antibodies and protocols were published previously for MPO [26] and CD3 [19]. The protocol for α-SMA was comparable to CD3. Primary antibodies were diluted 1:500, 1:1000 and 1:5000 for MPO, CD3 and α-SMA, respectively. For CD3, 0.3% H_2_O_2_ and 5% bovine serum albumin were used for blocking of endogenous peroxidase activity and for non-specific binding, respectively, while for α-SMA 0.5% H_2_O_2_ and 2% normal goat serum were used. Sections were stained with monoclonal mouse anti-α-SMA primary (A5228, Sigma-Aldrich) and polyclonal biotin-conjugated goat anti-mouse secondary antibodies (E0433; Dako, Glostrup, Denmark).

The number of CD3-positive cell infiltrates was counted within each lung section. An infiltrate was defined as at least ten CD3-positive cells per 2500 μm^2^. CD3-stained slides were scanned (magnification x200) with a VENTANA iScan HT slide scanner (Ventana Medical Systems, Inc.; Roche Group, Tucson, AZ). Next, the total lung parenchyma surface area was measured per slide using Pannoramic Viewer software (V1.15.4; 3DHISTECH Ltd., Budapest, Hungary). For each tissue section, the total number of CD3-positive cell infiltrates was divided by the total lung parenchyma surface area.

In addition, CD3-positive cells were counted in ten random representative images without infiltrates (magnification x200), corrected for the percentage of lung tissue per image using ImageJ software (ImageJ 1.45s, W. Rasband, US National Institutes of Health, Bethesda, MD) and averaged per animal.

Infiltration of MPO-positive cells was semi-quantitatively scored within the whole section as follows: 0, no cells; 1, minor number of cells; 2, medium number of cells; 3, large number of cells; and 4, very large number of cells.

All arterioles per α-SMA-stained lung section that met the same criteria as previously described [20] were measured. In short, all arterioles with <50 μm external diameter accompanying the terminal bronchiole (as identified by morphologic criteria) were measured. Only transversely sectioned blood vessels adjacent to the airways were analyzed to minimize distortion [20]. Morphometric measurement of the wall-to-lumen ratio (media thickness divided by the radius of the lumen) was performed on the digital images (magnification 400x) using Leica Qwin software (V3.5.1; Leica Microsystems, Wetzlar, Germany).

During the evaluation, counting and quantification of stained slides, all observers were blinded to experimental groups.

### Histology

For the detection of collagen, slides were incubated in 0.2% phosphomolybdic acid (HT153; Sigma-Aldrich) and stained for 90 minutes with 0.1% Sirius red (365548; Sigma-Aldrich) diluted in saturated picric acid (P6755, Sigma-Aldrich) in the dark. After incubation in 0.01N HCl, slides were dehydrated and coverslipped.

To assess adventitial fibrosis, all arterioles per lung section with the same criteria as previously described were semi-quantitatively scored by two blinded observers for the deposition of collagen around these arterioles: 1, mild; 2, moderate; 3, severe; and 4, very severe collagen deposition around arteriole [20].

### RNA extraction and real-time PCR

RNA extraction and real-time PCR were performed for the following genes: Angiopoietin-1 (*Ang1*), *VEGF*, VEGF receptor 2 (*VEGFR-2*), tunica interna endothelial cell kinase 2 (*Tie-2*) and ovine ribosomal protein S15 (*ovRPS15*). In brief, total RNA was extracted from snap-frozen RLL lung tissue by Trizol (15596018; Invitrogen, Life Technologies, Carlsbad, CA)/chloroform extraction. Isolated RNA was DNAse treated to remove possible contamination with genomic DNA by use of the RQ1 RNase-Free DNase kit (M6101; Promega, Madison, WI) and afterwards reverse transcribed into cDNA using oligo(dT)_12-18_ primers (18418–012; Invitrogen) and Moloney murine leukemia virus (M-MLV) reverse transcriptase (28025–021; Invitrogen). Real-time PCR reactions were performed in duplicate within a LightCycler 480 Instrument (Roche Applied Science, Basel, Switzerland) using the SensiMix™ SYBR® No-ROX Kit (QT650; Bioline, London, UK) for 45 cycles. Primer sequences (**Table 1**) were designed or used from literature [26, 27]. Results were normalized to the house keeping gene ovRPS15 and relative changes, over control values, were calculated.

**Table 1:** Primers used for Real-time PCR.

Gene		Sequence (5’-3’)	Reference
***Ang1***	Fw	TTGCCATAACCAGTCAGAG	[27]
	Rv	AACCACCAGCCTCCTGTTA	
***VEGF***	Fw	CATGCCAAGTGGTCCCAG	
	Rv	GAAGATGTCCACCAGGGTC	
***VEGFR-2***	Fw	CCCTGATTACACCACACC	
	Rv	TCTTTGCCATCCTGTTGAG	
***Tie-2***	Fw	CTATGGCGTGTTACTATGGGAG	
	Rv	GAGATCATACACCTCGTCGTC	
***ovRPS15***	Fw	CGAGATGGTGGGCAGCAT	[26]
	Rv	GCTTGATTTCCACCTGGTTGA	

Fw, forward; Rv, reverse; Ang1, Angiopoietin-1; VEGF, vascular endothelial growth factor; VEGFR-2, VEGF receptor 2; Tie-2, tunica interna endothelial cell kinase 2; ovRPS15, ovine ribosomal protein S15.

### Statistical analysis

GraphPad Prism v6.01 (GraphPad Software, La Jolla, CA) was used for statistical analysis. Experimental groups were compared using the non-parametric Kruskal-Wallis test, followed by Dunn’s Multiple Comparison Test for post-hoc analysis. All data are displayed as mean ± standard error of mean (SEM) and significance was considered as p<0.05.

## Results

### Pulmonary inflammation

**Fig 2:**
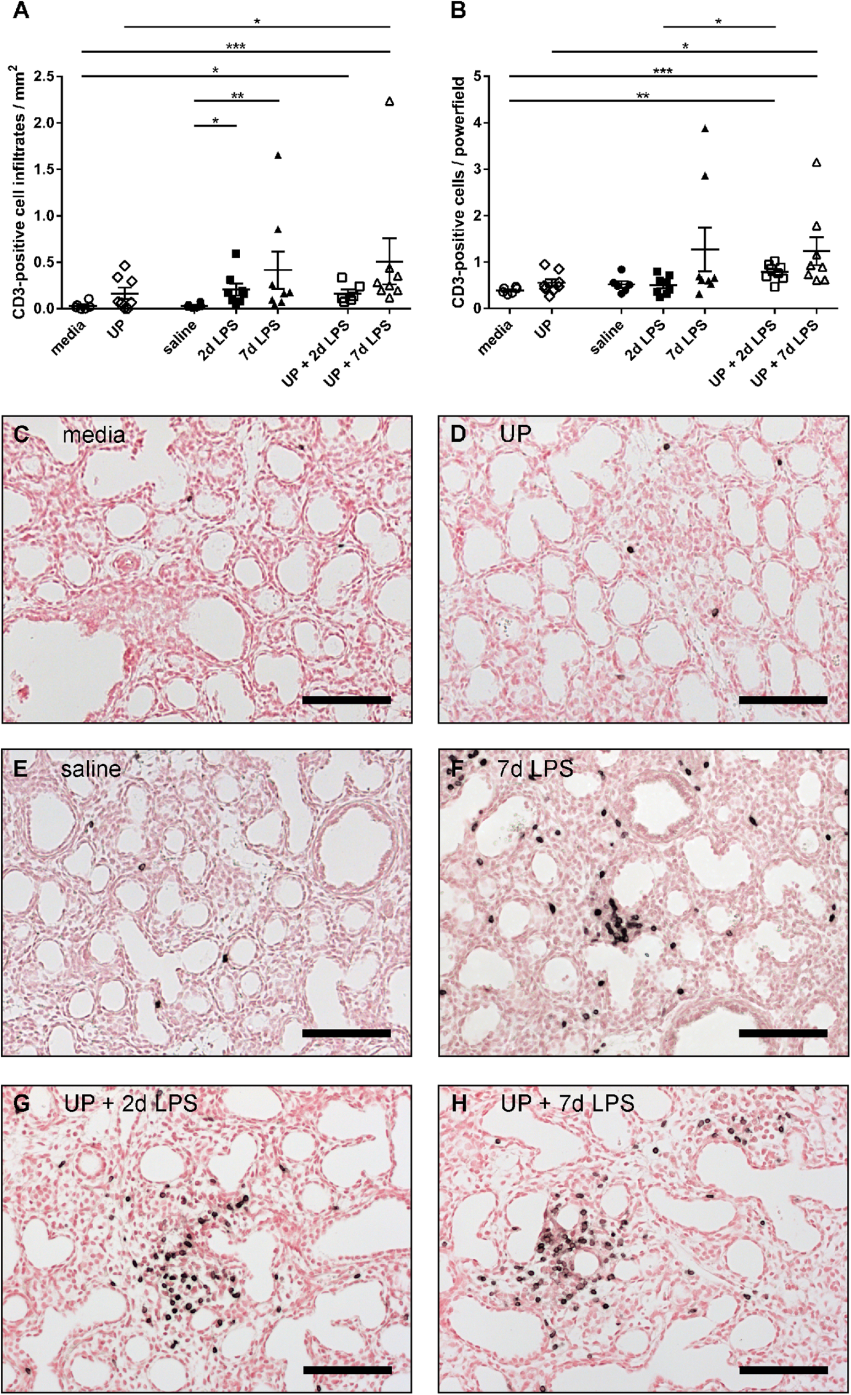
**CD3-positive cell infiltrate number (A) and quantification of CD3-positive cells (B) in preterm ovine lungs.** Representative pictures of lung sections, immunohistochemically stained for CD3, are shown for media- (C), *Ureaplasma parvum* (UP)- (D), saline- (E), 7d lipopolysaccharide (LPS)- (F), UP+2d LPS- (G) and UP+7d LPS-treated animals (H). Pictures show CD3-positive cell infiltrates (F-H) and singular CD3-positive cells not belonging to cell infiltrates (C-E). Scale bars: 100 μm. *p<0.05, **p<0.01 and ***p<0.001 between experimental groups using the non-parametric Kruskal-Wallis test, followed by Dunn’s Multiple Comparison Test for post-hoc analysis.

First, we addressed whether the extreme premature lung mounted an inflammatory response following an intra-amniotic inflammatory stimulus. We observed that CD3-positive cells specifically clustered into infiltrates around vascular and bronchiolar structures after exposure to an intra-amniotic inflammatory stimulus. Therefore, we quantified the number of CD3-positive cell infiltrates in the ovine fetal lungs (**Fig 2A**) and found that chronic *U*. *parvum* exposure did not significantly raise the infiltrate number compared with the media group. The CD3-positive cell infiltrate count increased in the 2d LPS- and 7d LPS-treated animals compared with the saline group (**Fig 2A, 2E and 2F**). Infiltrate numbers were also increased in the UP+2d LPS and UP+7d LPS groups compared with UP- and/or media-treated animals (**Fig 2A, 2C, 2D, 2G and 2H**). Furthermore, CD3-positive cell numbers were quantified in representative lung pictures without infiltrates, to assess whether the overall spread of CD3-positive cells within the lung tissue was different between experimental groups. No differences were found between UP- and media-treated animals (**Fig 2B–2D**). CD3-positive cell numbers were also elevated in the UP+2d LPS and UP+7d LPS groups compared with UP- and/or media-treated animals, which is in line with the CD3-positive cell infiltrates of **Fig 2A**. In addition, the UP+2d LPS group showed higher CD3-positive cell numbers compared to the 2d LPS group. These combined data suggest an overall increase in the number of CD3-positive cells after exposure to LPS alone or to the combination of *U*. *parvum* with LPS compared with control animals (media and saline groups).

**Fig 3:**
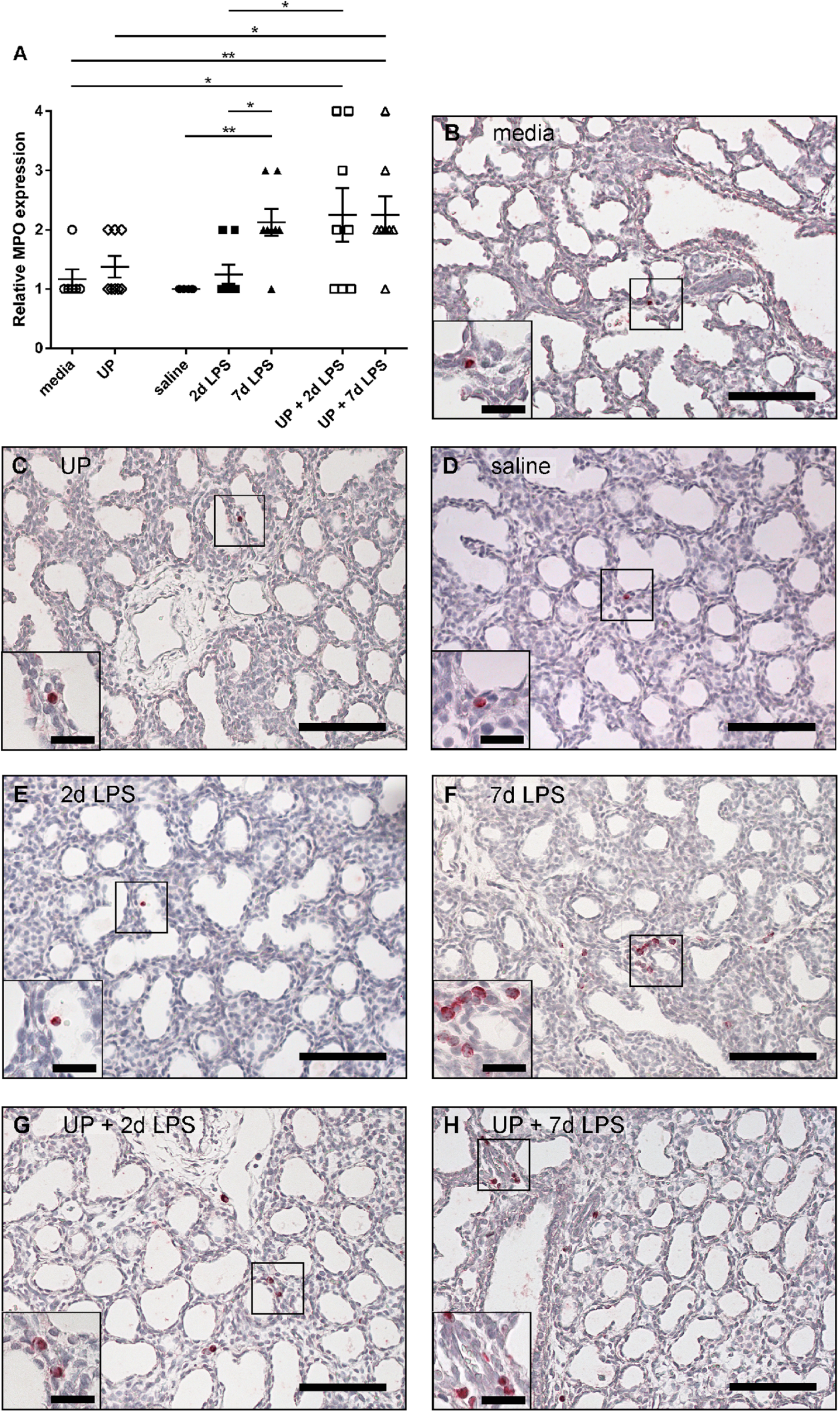
Semi-quantitative scoring of myeloperoxidase (MPO)-positive cells in the extreme preterm ovine lung. Lung sections were immunohistochemically stained for MPO. Representative images of MPO-positive cells are shown for media- (B), *Ureasplasma parvum* (UP)- (C), saline- (D), 2d lipopolysaccharide (LPS)- (E), 7d LPS- (F), UP+2d LPS- (G) and UP+7d LPS-treated (H) animals. Inserts in the left lower corner show a higher magnification of the squared area. Scale bars: 100 μm; scale bars in inserts: 25 μm. *p<0.05 and **p<0.01 between experimental groups, analyzed with the non-parametric Kruskal-Wallis test and Dunn’s Multiple Comparison Test for post-hoc analysis.

Intra-amniotic administration of *U*. *parvum* did not affect the amount of MPO-positive cells in the pulmonary tissue compared with the media group (**Fig 3A–3C**). The amount of MPO-positive cells did not change within the 2d LPS group, while exposure to 7 days of LPS increased the accumulation of MPO-positive cells compared with the saline and 2d LPS groups (**Fig 3A, 3D–3F**). MPO-positive cell infiltration was enhanced in the UP+2d LPS group compared with media- and 2d LPS- treated animals (**Fig 3A, 3B, 3E and 3G**). UP+7d LPS-treated animals had an increased amount of MPO-positive cells compared with media- and UP-treated animals (**Fig 3A–3C and 3H**).

### Pulmonary arteriolar remodeling

**Fig 4:**
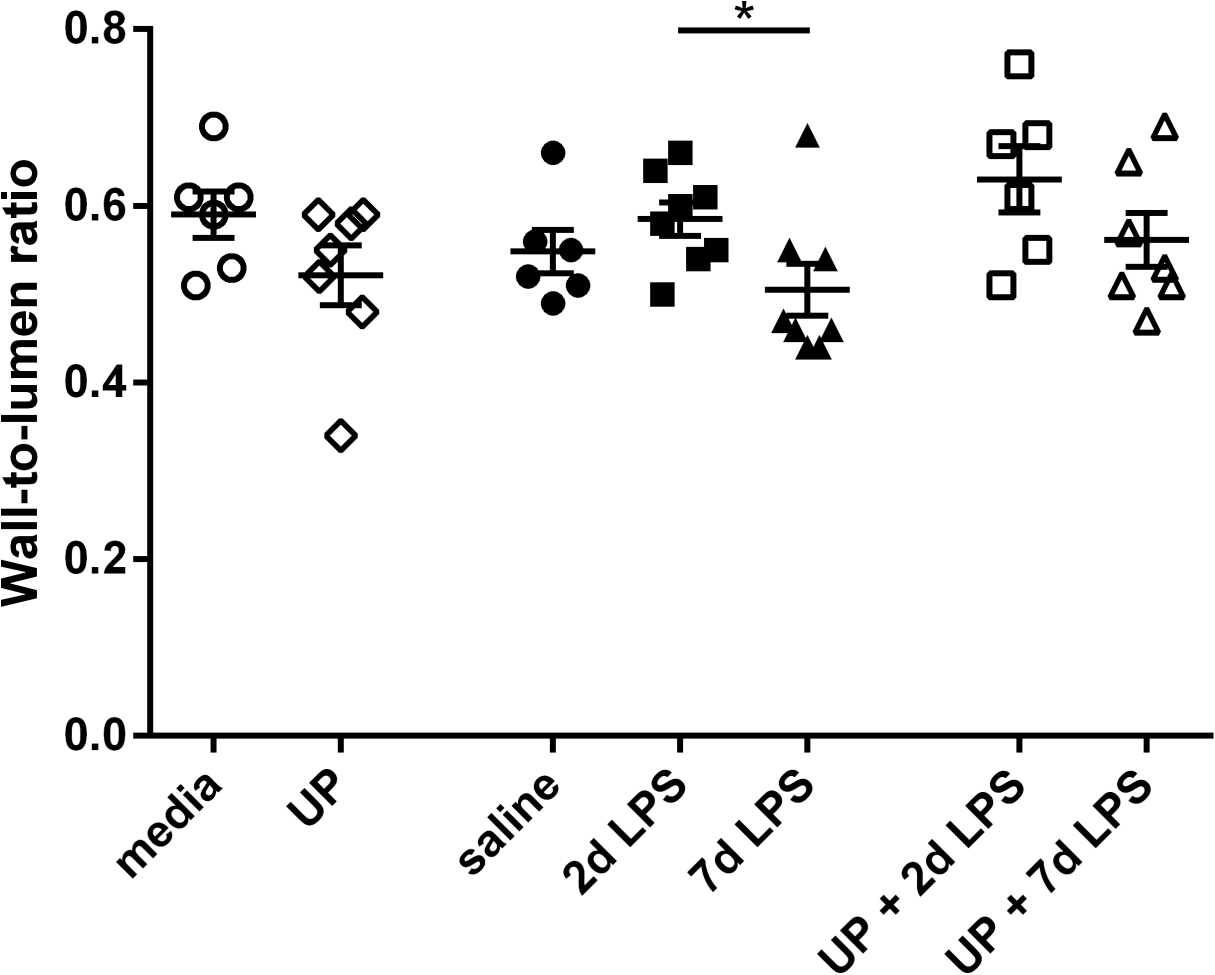
Wall-to-lumen ratio of pulmonary arterioles in *Ureaplasma parvum* (UP)- and/or lipopolysaccharide (LPS)-treated extreme preterm sheep. *p<0.05 between experimental groups.

Since antenatal inflammation is known to induce vascular remodeling of small pulmonary arteries in the preterm lung at 122 dGA [20], we determined whether intra-amniotic exposure to *U*. *parvum* and/or LPS at an extreme preterm age also led to vascular remodeling. Vascular remodeling is defined as a structural change in the lumen diameter which can result in a changed wall-to-lumen ratio [28, 29]. To assess vascular remodeling, we therefore defined the wall-to-lumen ratio of pulmonary arterioles. The ratio was unchanged in the UP group compared with the media group (**Fig 4**). Animals exposed to LPS for 7 days had a decreased wall-to-lumen ratio compared with 2d LPS-treated animals. Compared with single exposure to UP alone, combined exposure to UP+2d LPS increased the wall-to-lumen ratio with approaching significance (p = 0.06). Within the UP+7d LPS group, the ratio was unaffected.

**Fig 5:**
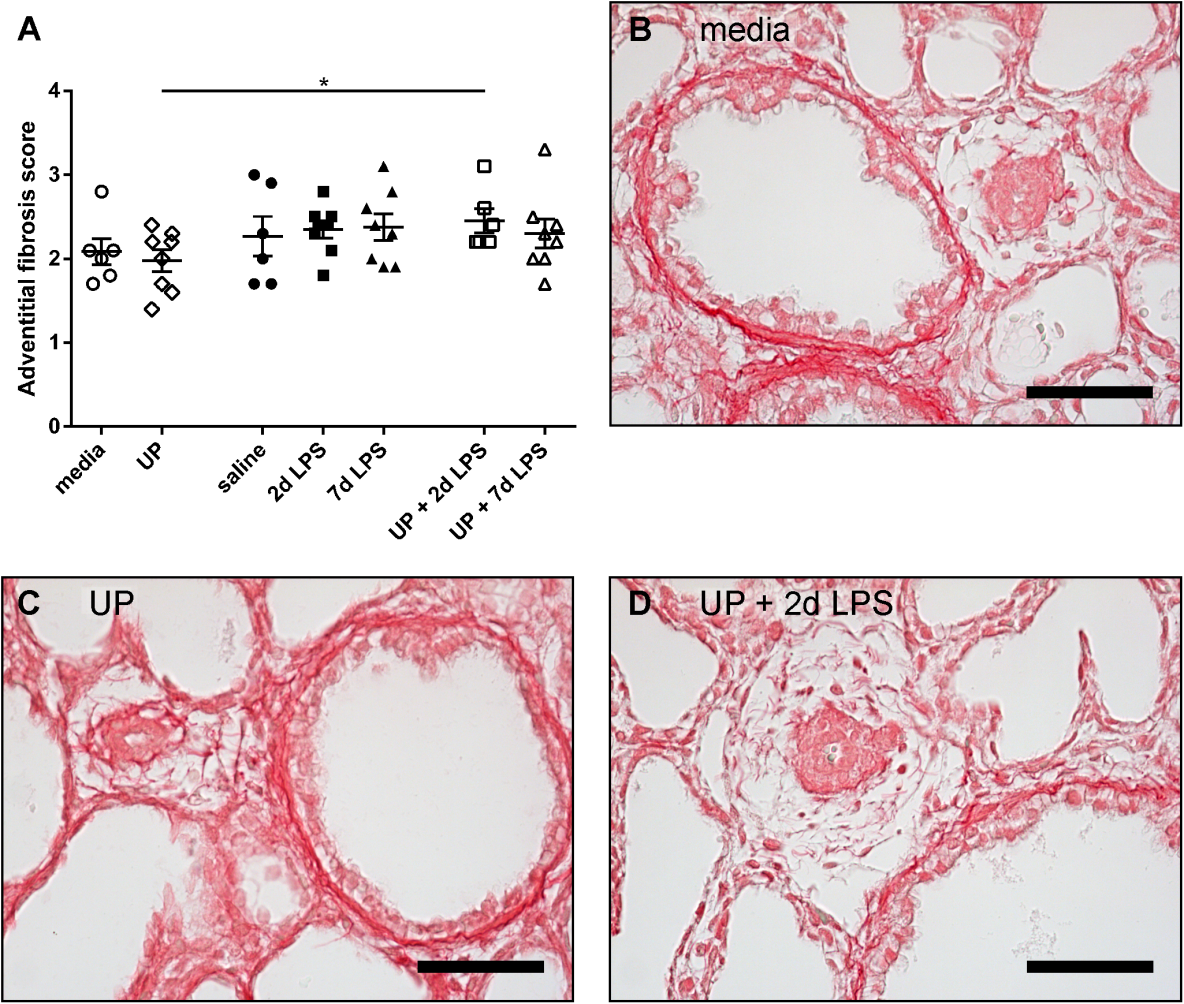
Adventitial fibrosis score of pulmonary arterioles in *Ureaplasma parvum* (UP)- and/or lipopolysaccharide (LPS)-treated preterm sheep. Representative pictures of lung sections histologically stained for collagen are shown for media- (B), UP- (C) and UP+2d LPS-treated animals (D). Scale bars: 50 μm. *p<0.05 between experimental groups using the non-parametric Kruskal-Wallis test, followed by Dunn’s Multiple Comparison Test for post-hoc analysis.

Furthermore, we assessed the accumulation of collagen in the adventitia of pulmonary arterioles, as adventitial matrix deposition is common during pulmonary artery remodeling [30]. Although a single exposure to either UP, 2d LPS or 7d of LPS had no effect compared with control animals, dual exposure to UP+2d LPS increased the adventitial fibrosis score compared with single exposure to UP (**Fig 5A–5D**). No effect on adventitial fibrosis was found in the UP+7d LPS group.

### Expression of angiogenic growth factors & receptors

**Fig 6:**
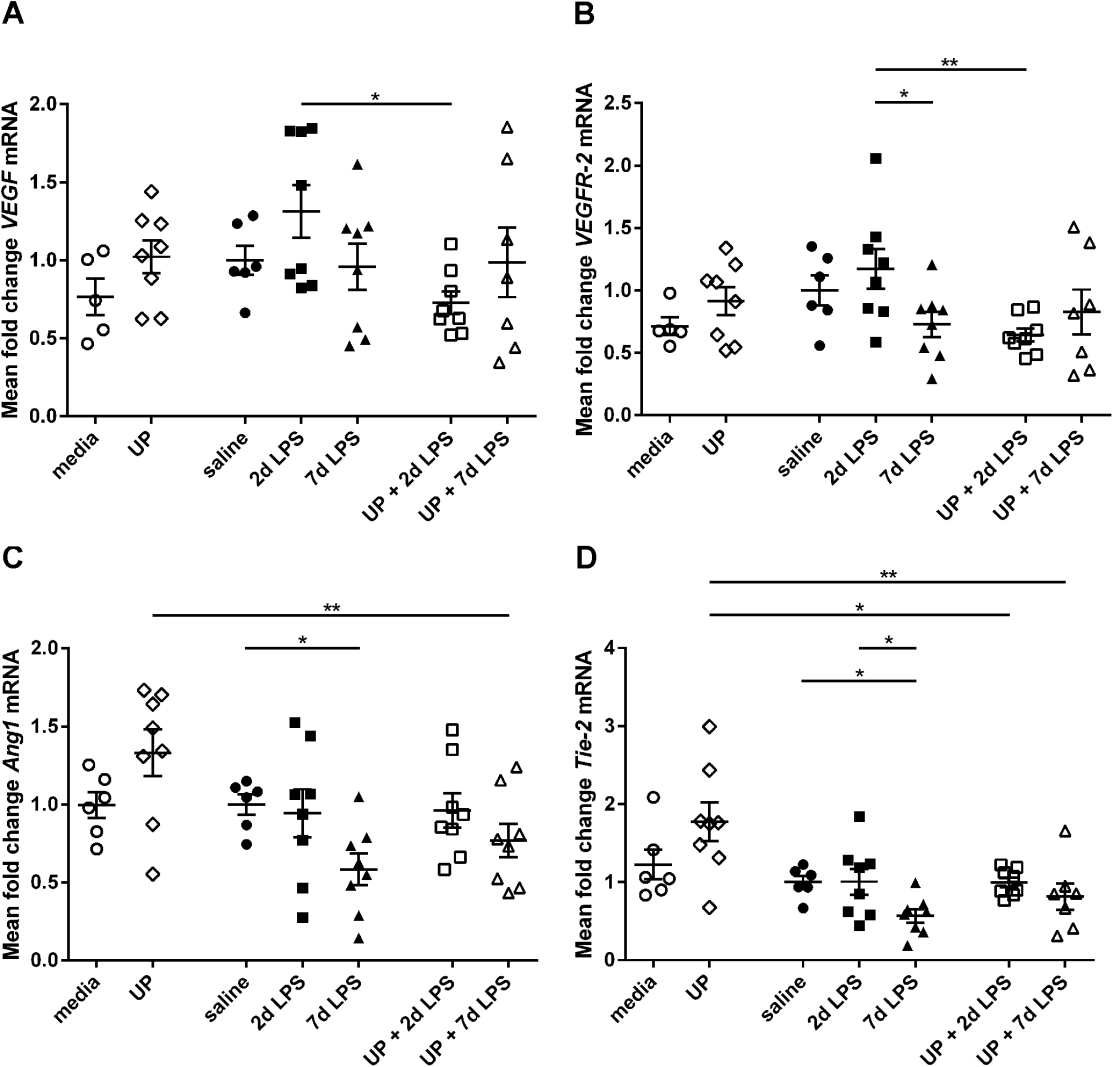
Relative mRNA levels of angiogenic growth factors and receptors in the extreme preterm ovine lung. Pulmonary mRNA levels of vascular endothelial growth factor (*VEGF*) (A), VEGF receptor 2 (*VEGFR-2*) (B), Angiopoietin-1 (*Ang1*) (C) and tunica interna endothelial cell kinase 2 (*Tie-2*) (D) are shown for *Ureaplasma parvum* (UP)- and/or lipopolysaccharide (LPS)-treated preterm sheep. Results were obtained using real-time PCR reactions. Data were normalized to the house keeping gene ovRPS15. *p<0.05 and **p<0.01 between experimental groups with the non-parametric Kruskal-Wallis test and Dunn’s Multiple Comparison Test for post-hoc analysis.

Since VEGF and VEGF receptors are essential for pulmonary vascular growth [12, 13], we determined lung mRNA levels of *VEGF* and *VEGFR-2* after antenatal inflammation. Intra-amniotic exposure to *U*. *parvum* or LPS alone did not affect *VEGF* mRNA levels compared with control animals (**Fig 6A**). Dual exposure to UP+2d LPS decreased *VEGF* mRNA levels compared with single exposure to 2d LPS. Similar results were found for *VEGFR-2* mRNA levels (**Fig 6B**). Intra-amniotic administration of *U*. *parvum* did not affect *VEGFR-2* mRNA levels, while *VEGFR-2* levels were decreased after combined exposure to UP+2d LPS compared with single exposure to 2d LPS. In addition, exposure to LPS for 7 days decreased *VEGFR-2* mRNA levels compared with exposure to 2 days of LPS.

Since Angiopoietin-1 and its receptor Tie-2 are necessary for vessel maturation and stability [9, 13], their expression levels were determined in the ovine fetal lungs. *Ang1* mRNA levels were unchanged in the UP group, compared with the media group (**Fig 6C**). 7d LPS-treated animals had reduced *Ang1* mRNA levels compared with saline animals (**Fig 6C**). Reduced *Ang1* mRNA levels were also found in UP+7d LPS exposed animals, when compared with UP-treated animals. Similar findings were observed for *Tie-2* mRNA levels (**Fig 6D**). *Tie-2* mRNA levels remained unaltered after exposure to *U*. *parvum*, while exposure to 7d of LPS attenuated *Tie-2* levels. UP+2d LPS- and UP+7d LPS-treated animals had decreased *Tie-2* mRNA levels compared with the UP group.

## Discussion

Since the incidence of histologic chorioamnionitis is inversely correlated with gestational age at preterm birth and because extremely preterm infants are often born during critical stages of pulmonary vascular development [6, 10, 23, 24], we evaluated in this study whether exposure to intra-uterine inflammatory stimuli resulted in pulmonary inflammation and vascular changes in extreme premature fetuses at the canalicular stage.

Preterm infants have an immature immune system which may be further compromised by additional factors related to preterm birth [31–33]. Whereas our group previously demonstrated that intra-amniotic LPS stimulated fetal lung and skin inflammation in extreme preterm sheep [34], we determined in this study how the extremely premature sheep lung responded to chronic *U*. *parvum* exposure. Contrary to LPS, chronic exposure to *U*. *parvum* did not provoke a pulmonary inflammatory response. Previous research with ovine fetuses born at a more mature age already indicated that acute or chronic *U*. *parvum* exposure was only responsible for mild lung inflammation due to low virulence [19, 21].

Previously, it was demonstrated that chronic intra-amniotic pre-exposure to *U*. *parvum* decreased the pulmonary immune response to subsequent contact with LPS, suggesting that chronic intra-uterine inflammation attenuates fetal immune responses and thereby increases the risk for a following nosocomial infection [21]. Postnatal nosocomial infections are quite common in preterm infants due to extended stay within a hospital [31]. Nevertheless, our early gestational age study indicates that chronic exposure to *U*. *parvum* did not diminish subsequent LPS responsiveness within the fetal lungs. The presence of CD3- and MPO-positive cells even increased after dual *U*. *parvum* and LPS exposure compared with 2d LPS or *U*. *parvum* alone. Levy et al. [35] and Netea et al. [36] already suggested that stimulation of the innate immune system of the neonate may alter the innate immunity set point and leads to immunological memory of the innate response (so-called ‘trained immunity’). This may result in a heightened immune response towards a following challenge with the same or a different microorganism [35, 36]. Although trained immunity may be beneficial for the vulnerable neonate while adaptive immune responses are still limited, it may also be harmful in the sense that enhanced immune responses can result in concomitant neonatal morbidities [37]. Our study and that of Kallapur et al. [21] furthermore suggest that the gestational age at exposure to the first stimulus (chronic *U*. *parvum*) as well as the gestational age at preterm birth are both important and clearly influence the pulmonary inflammatory response and the response to a second inflammatory stimulus.

Structural changes in adventitial fibrosis score after *U*. *parvum* plus 2d LPS versus *U*. *parvum* were associated with significant changes in Tie2 mRNA levels. The significant changes in VEGF-R2 and Tie2 mRNA levels between 2d LPS and 7d LPS exposed animals were paralleled by a difference in the wall to lumen. There were however no significant structural changes between the media/saline animals and the UP and/or LPS exposed animals despite significant differences in Ang1 and Tie2 mRNA levels between the saline and 7d LPS group. It is tempting to speculate that these latter changes will be followed by subsequent structural and functional changes as previously shown in sheep and rat models of LPS chorioamnionitis[20, 38]. In addition, human studies indicated that deceased BPD patients had decreased *VEGF* and *Tie-2* mRNA levels within their lungs [9]. In the pathophysiology of BPD it is thought that numerous prenatal (such as chorioamnionitis) and postnatal factors can impair angiogenic signaling, which disrupts vascular growth and causes abnormal vascular function. Increased pulmonary vascular resistance can alter vasoreactivity and causes vascular remodeling. All these steps may eventually contribute to the development of pulmonary hypertension in the setting of BPD [39]. Therefore, the disturbed expression of angiogenic factors as observed in our study may potentially contribute to the pathogenesis of BPD and BPD-associated pulmonary hypertension.

According to the vascular hypothesis, disrupted pulmonary vascular development during essential stages of development can lead to impaired alveolarization and contributes to pulmonary hypoplasia [10, 11]. In neonatal rats it was already revealed that anti-angiogenic agents not only reduced pulmonary vascular growth but also impaired alveolarization [12, 13]. We were not able to assess parameters involved with alveolarization, due to the fact that alveolar development has not yet started at 94 dGA [22]. In addition, pulmonary function could not be evaluated, since the lungs were too fragile at this stage. Therefore, we can only speculate about the consequences of the observed vascular changes for the later alveolar development in our model. Previously, chronic endotoxin exposure starting at 80 dGA and using an intra-amniotic osmotic pump, resulted in decreased vascular proteins and a reduced saccular number within the ovine fetal lungs at 100 dGA. However, within the same model no residual vascular and morphometric effects of this chronic endotoxin exposure were observed at 138 dGA [40]. A second chronic exposure model using multiple endotoxin injections from 100 dGA onwards caused small vascular changes in sheep delivered at 130 dGA or 145 dGA (near-term) and tended to lower alveolar numbers with 30% in the 145 dGA animals [40]. Kallapur et al. [40] postulated that the ovine fetal lungs recover and develop rather normal in spite of exposure to chronic endotoxin, when examined closer to term. Nevertheless, the study designs of these two chronic endotoxin exposure studies [40] are different from ours considering timing and model of exposure and the age at preterm birth. Therefore, further research is needed to explore whether vascular changes at an extreme preterm age (94 dGA), as induced by exposure to single or combined intra-amniotic *U*. *parvum* and LPS, will remain and will be accompanied by impaired alveolar development at an older gestational age (e.g. near-term) or after exposure to additional postnatal inflammatory stimuli.

In conclusion, we have shown that contamination of the amniotic fluid is capable to mount an inflammatory response in the lungs of extreme preterm fetuses with concomitant impairment of the vasculature. Chronic pre-exposure to *U*. *parvum* and subsequent LPS enhanced the pulmonary inflammatory response compared to exposure with LPS or *U*. *parvum* alone. In addition, combined exposure to *U*. *parvum* and LPS resulted in disturbed expression of angiogenic growth factors and receptors compared with single exposure to *U*. *parvum* or LPS. This study contributes to our understanding regarding the effects of antenatal inflammation on vascular lung development, which is important for normal pulmonary development. Consequently, modulation of vascular development may be potentially beneficial in the treatment of inflammation-associated lung diseases, such as BPD.
